# The relationship between nutritional status and sleep quality in Parkinson’s disease: a single tertiary center study

**DOI:** 10.3389/fneur.2025.1731273

**Published:** 2026-01-02

**Authors:** Tuanfeng Yang, Jia Lu, Kehan Jin, Xinyue Jiang, Xianzeng Liu

**Affiliations:** 1 Department of Neurology, Peking University International Hospital, Beijing, China; 2 Clinical Research Institute, Institute of Advanced Clinical Medicine, Peking University, Beijing, China

**Keywords:** malnutrition, malnutrition risk, MNA, Parkinson’s disease, PDSS, sleep disturbances

## Abstract

**Objective:**

To investigate the prevalence of malnutrition, sleep disturbances, and the relationship between nutritional status and sleep quality in patients with Parkinson’s disease (PD).

**Methods:**

The study included 168 PD patients and 102 Healthy Controls (HCs) from January 2019 to December 2024 in Peking University International Hospital. Mini Nutritional Assessment (MNA) was used to determine malnutrition and risk of malnutrition. Sleep quality was assessed by Parkinson’s Disease Sleep Scale (PDSS). Hoehn-Yahr (H-Y) stage and Unified Parkinson’s Disease Rating Scale Part III (UPDRSIII) were used to assess the motor severity of PD. Nutritional biomarkers, such as body mass index (BMI), hemoglobin, neutrophil-to-lymphocyte ratio (NLR), total protein, albumin, prealbumin, uric acid, total cholesterol (TC), triglyceride (TG), high-density lipoprotein cholesterol (HDL-C), low-density lipoprotein cholesterol (LDL-C), homocysteine, vitamin B12, ferritin, folate and glycosylated hemoglobin (HbA1c) were included. The relationship between nutritional status and sleep quality was analyzed.

**Results:**

The study found the prevalence of malnutrition and malnutrition risk were 10.1 and 17.9% respectively, while 52.3% patients suffered from sleep disturbances in PD. There was significant difference between PD patients with sleep disturbances and those without in age, duration, H-Y stage, UPDRSIII, MNA, BMI, hemoglobin, total protein, albumin and prealbumin (all *P* < 0.05). Logistic regression analysis preliminary identified H-Y stage and total protein as significant risk factors for sleep disturbances in PD patients (*P* < 0.05).

**Conclusion:**

The study suggested malnutrition and malnutrition risk, and sleep disturbances were prevalent in PD patients. Nutritional biomarkers such as total protein are closely related to sleep quality in PD patients.

## 1 Introduction

Parkinson’s disease (PD) is the second most prevalent neurodegenerative disorder after Alzheimer’s disease, characterized by progressive degeneration of dopaminergic neurons in the substantia nigra. The all age prevalence of PD is approximately 152 per 100,000 in general populations according to the Global Burden of Disease 2021 data (1), while in China, it affects about 1.7% of those aged 65 and older (2). Traditionally viewed as a predominantly motor disorder, PD manifests with core motor features including bradykinesia, rigidity, resting tremor, and postural instability. However, non-motor symptoms are increasingly recognized as integral components of the disease and include sleep disturbances, sensory impairments, autonomic dysfunctions, neuropsychiatric manifestations, as well as unintended weight loss (3). These non-motor complications significantly contribute to increased disease burden and diminished quality of life for both patients and caregivers.

Malnutrition and weight loss are more prevalent among PD patients compared to age-matched controls without the disease (4–6). A systematic review reported that the prevalence of malnutrition in PD ranges from 0 to 24%, with an additional 3 to 60% of patients at risk of malnutrition (4). A range of factors, including increased energy expenditure, gastrointestinal dysfunction, anxiety, and levodopa-related side effects, contribute to an elevated risk of malnutrition (4–7). Sleep disturbances are also highly prevalent, affecting between 60 and 98% of PD patients (5). These disturbances encompass a broad range of sleep disorders, including insomnia, excessive daytime sleepiness, sleep-related breathing disorders, circadian rhythm disruptions, sleep-related movement disorders such as restless legs syndrome (RLS) and periodic limb movements during sleep (PLMS), and parasomnias, particularly rapid eye movement sleep behavior disorder (RBD) (8–10). In some cases, malnutrition and sleep disturbances may cause morbidity comparable to or even exceeding that of motor symptoms, substantially impairing daily functioning and overall well-being. Despite their high frequency and clinical significance, these issues are frequently underrecognized and undertreated in clinical practice. Nutritional and sleep assessments remain inconsistently integrated into the routine evaluation and management of PD patients in many healthcare settings.

A bidirectional relationship has been established between malnutrition and sleep disturbances in older adults, wherein each condition can exacerbate the other (11). However, research exploring this association specifically within the PD population remains limited (12). This study aims to investigate the relationship between nutritional status and sleep disturbances among PD patients in a single tertiary care center.

## 2 Methods

### 2.1 Subjects

A total of 168 patients with PD and 102 age- and sex-matched healthy controls (HCs) were enrolled at Peking University International Hospital between January 2019 and December 2024. The diagnosis of PD was established according to the clinical diagnostic criteria of the Movement Disorder Society (13). Exclusion criteria included evidence of secondary parkinsonism or Parkinson-plus syndromes; a history of malignant tumors, hyperthyroidism, confirmed structural gastrointestinal disorders, acute infection, or autoimmune diseases; taking melatonin, antipsychotics, or antidepressants; and inability to cooperate with physical examination or complete required questionnaires. Written informed consent was obtained from all participants, including both PD patients and healthy controls. The study was approved by the Ethics Committee of Peking University International Hospital [2021-052(BMR)].

### 2.2 Clinical features and assessments

For each PD patient, the following data were collected: age, age at disease onset, disease duration, primary motor symptoms, disease progression, medical history, concomitant medications, comorbidities, and neurological examination findings. For HCs, age, medical history, and existing complications were recorded. Additional information was extracted from electronic medical records.

All questionnaire assessments were conducted by a trained researcher who had completed standardized training prior to data collection. Face-to-face interviews were performed with participants and their caregivers. PD patients were assessed during their “on” state. The levodopa equivalent dose (LED) was calculated using established conversion formulas (14). Motor severity was evaluated using the Hoehn-Yahr (H-Y) stage (15) and the Unified Parkinson’s Disease Rating Scale Part III (UPDRSIII) (16).

The Mini Nutritional Assessment (MNA) is a validated tool designed to evaluate nutritional status in individuals aged 65 years and older, consisting of 18 items that include anthropometric measurements such as body mass index (BMI), mid-arm circumference, and calf circumference (17). BMI was calculated as weight in kilograms divided by height in meters squared. The MNA is recommended for the early identification of malnutrition risk, particularly in individuals with normal albumin levels and BMI. The maximum total score is 30, with higher scores indicating better nutritional status. A score of 24–30 indicates adequate nutrition, 17–23.5 indicates risk of malnutrition, and a score below 17 indicates malnutrition.

The Parkinson’s Disease Sleep Scale (PDSS) is a self-reported visual analogue scale assessing 15 common sleep-related symptoms experienced over the preceding week (18). It evaluates the following domains: overall sleep quality (item 1), sleep onset and maintenance insomnia (items 2 and 3), nocturnal restlessness (items 4 and 5), nocturnal psychosis (items 6 and 7), nocturia (items 8 and 9), nocturnal motor symptoms (items 10–13), sleep refreshment (item 14), and daytime somnolence (item 15). Participants rated each item on a 10-cm visual analogue scale ranging from worst (0) to best (10) condition. The total score is the sum of all item scores, with a maximum of 150. A total score of ≤82 was defined as indicative of poor sleep quality, based on previous research (19).

Blood samples were collected from all participants on the morning following admission, after a minimum 10-h fast without food or fluid intake. Fasting blood was analyzed for a panel of nutritional biomarkers, including hemoglobin, neutrophil-to-lymphocyte ratio (NLR), total protein, albumin, prealbumin, uric acid, total cholesterol (TC), triglycerides (TG), high-density lipoprotein cholesterol (HDL-C), low-density lipoprotein cholesterol (LDL-C), homocysteine, vitamin B12, ferritin, folate, and glycosylated hemoglobin (HbA1c).

### 2.3 Statistical analysis

Statistical analyses were conducted using SPSS software (version 17.0). The Kolmogorov–Smirnov test was employed to assess the normality of distribution for continuous variables. Normally distributed data are presented as mean ± standard deviation, whereas non-normally distributed variables, including duration and H-Y stage, are reported as median and interquartile range (25th-75th percentiles). Group comparisons between two independent samples were performed using either the Student *t* test or the Mann–Whitney U test, depending on the distribution of the data. Categorical variables are expressed as frequency and percentage (*n*, %), and the Chi-square test was used for intergroup comparisons. Multivariate logistic regression analysis was utilized to examine the association between nutritional markers and sleep disturbances. A *p*-value of less than 0.05 was considered statistically significant.

## 3 Results

Tables 1, 2 show the comparison of clinical features and nutritional biomarkers between PD and HCs groups. 168 PD patients and 102 HCs were included in the study. There was no significant difference in terms of sex and age between the two groups (all P>0.05). In PD group, we rated 10 cases as H-Y stage 1, 5 as stage 1.5, 59 as stage 2, 30 as stage 2.5, 44 as stage 3, 18 as stage 4, and 2 as stage 5 (Table 3). According to the MNA score, 17 cases (10.1%) were classified as malnutrition, and 30 cases (17.9%) were at risk of malnutrition in PD. None was classified as malnutrition, and 8 cases (7.8%) were at risk of malnutrition in HCs. Sleep disturbances occurred in 88 cases (52.3%) in PD, and in 16 cases (15.7%) in HCs based on the PDSS score. Compared with HCs, PD patients showed lower PDSS score, MNA score, BMI, hemoglobin, total protein, albumin, uric acid, TG, LDL-C and ferritin, and higher HCY (all *p* < 0.05).

**Table 1:** Comparison of clinical features between PD and HCs groups.

Feature	PD group (*n* = 168)	HCs group (*n* = 102)	*P*
Male (*n*)	93	60	0.577#
Age (year)	79.76 ± 8.42	67.83 ± 8.02	0.064*
MNA	24.15 ± 3.93	27.07 ± 2.43	<0.001*
PDSS	88.50 ± 24.38	111.80 ± 19.96	<0.001*
Duration (year)	2 (1, 4)	NA	NA
UPDRSIII	27.46 ± 14.52	NA	NA
LED (mg)	322.43 ± 200.80	NA	NA

MNA, Mini Nutritional Assessment; PDSS, Parkinson’s Disease Sleep Scale; UPDRSIII, Unified Parkinson’s Disease Rating Scale Part III; LED, levodopa equivalent dose; NA, not applicable. # Chi-square test; * Student *t* test.

**Table 2:** Comparison of nutritional biomarkers between PD and HCs groups.

Characteristic	PD group (*n* = 168)	HCs group (*n* = 102)	*P*
BMI	23.55 ± 2.81	24.93 ± 3.13	<0.001*
Hemoglobin (g/L)	131.48 ± 13.49	138.75 ± 15.55	<0.001*
NLR	2.37 ± 1.14	2.44 ± 1.42	0.622*
Total protein (g/L)	64.53 ± 5.26	67.73 ± 4.26	<0.001*
Albumin (g/L)	39.00 ± 3.42	41.69 ± 3.20	<0.001*
Prealbumin (mg/L)	234.63 ± 52.04	230.06 ± 45.46	0.464*
Uric acid (umol/L)	308.11 ± 79.13	329.70 ± 76.31	0.029*
TC (mmol/L)	4.05 ± 0.98	4.20 ± 0.99	0.216*
TG (mmol/L)	1.30 ± 0.88	1.79 ± 1.49	0.003*
HDL-C (mmol/L)	1.15 ± 0.28	1.13 ± 0.27	0.612*
LDL-C (mmol/L)	2.33 ± 0.77	2.57 ± 0.68	0.013*
homocysteine (umol/L)	15.07 ± 5.51	12.70 ± 3.93	<0.001*
Vitamin B12 (pg/ml)	490.88 ± 320.25	517.67 ± 371.37	0.531*
Ferritin (ng/ml)	156.62 ± 106.65	219.86 ± 151.09	<0.001*
Folate (ng/ml)	6.85 ± 3.84	7.69 ± 3.89	0.082*
HbA1c (%)	6.24 ± 0.94	6.39 ± 1.25	0.253*

MNA, Mini Nutritional Assessment; PDSS, Parkinson’s Disease Sleep Scale; BMI, body mass index; NLR, neutrophil-to-lymphocyte ratio; TC, total cholesterol; TG, triglyceride; HDL-C, high-density lipoprotein cholesterol; LDL-C, low-density lipoprotein cholesterol; HbA1c glycosylated hemoglobin. * Student *t* test.

**Table 3:** PDSS data in PD patients with different H-Y stages.

H-Y stage	*N*	PDSS
1	10	130.00 ± 2.49
1.5	5	123.40 ± 4.56
2	59	96.90 ± 20.83
2.5	30	86.83 ± 16.86
3	44	79.55 ± 17.48
4	18	57.00 ± 7.13
5	2	51.50 ± 2.12

H-Y stage, Hoehn-Yahr stage; PDSS, Parkinson’s Disease Sleep Scale.

Table 4 shows subgroup comparison of clinical features and nutritional biomarkers between poor nutrition and normal nutrition groups in PD. 47 (28.0%) PD patients demonstrated poor nutrition including those with malnutrition and at risk of malnutrition. Compared with normal nutrition group, the poor nutrition group showed longer duration, higher H-Y stage, UPDRSIII score, and NLR, lower PDSS score, BMI, hemoglobin, total protein, albumin, prealbumin and TG (all *p* < 0.05).

**Table 4:** Subgroup comparison of clinical features and nutritional biomarkers between poor nutrition and normal nutrition groups in PD.

Characteristic	Poor nutrition group (*n* = 47)	Normal nutrition group (*n* = 121)	*P*
Male (*n*)	28	65	0.493#
Age (year)	72.64 ± 7.74	68.64 ± 8.43	0.005*
Duration (year)	3 (2, 4)	2 (1, 4)	0.024**
H-Y stage	3 (2, 4)	2.5 (2, 3)	<0.001**
UPDRSIII	35.30 ± 18.42	24.42 ± 11.40	<0.001*
LED (mg)	340.63 ± 196.34	315.57 ± 202.95	0.503*
MNA	18.62 ± 3.11	26.31 ± 1.26	<0.001*
PDSS	72.70 ± 19.39	94.64 ± 23.39	<0.001*
BMI	21.07 ± 2.46	24.52 ± 2.30	<0.001*
Hemoglobin (g/L)	126.68 ± 14.05	133.34 ± 12.86	0.004*
NLR	2.83 ± 1.61	2.19 ± 0.85	0.011*
Total protein (g/L)	62.64 ± 6.02	65.27 ± 4.76	0.003*
Albumin (g/L)	36.12 ± 3.03	40.12 ± 2.87	<0.001*
Prealbumin (mg/L)	207.30 ± 59.33	245.25 ± 44.87	<0.001*
Uric acid (umol/L)	292.21 ± 78.90	314.29 ± 78.67	0.105*
TC (mmol/L)	4.08 ± 0.80	4.04 ± 1.05	0.842*
TG (mmol/L)	1.03 ± 0.57	1.40 ± 0.96	0.014*
HDL-C (mmol/L)	1.21 ± 0.32	1.13 ± 0.27	0.076*
LDL-C (mmol/L)	2.26 ± 0.63	2.36 ± 0.83	0.380*
homocysteine (umol/L)	15.19 ± 5.86	15.02 ± 5.40	0.861*
Vitamin B12 (pg/ml)	587.11 ± 403.72	453.50 ± 274.26	0.051*
Ferritin (ng/ml)	179.48 ± 141.02	147.74 ± 89.00	0.156*
Folate (ng/ml)	7.53 ± 4.46	6.58 ± 3.55	0.151*
HbA1c (%)	6.06 ± 0.98	6.31 ± 0.91	0.130*

H-Y stage, Hoehn-Yahr stage; UPDRSIII, Unified Parkinson’s Disease Rating Scale Part III; LED, levodopa equivalent dose; MNA, Mini Nutritional Assessment; PDSS, Parkinson’s Disease Sleep Scale; BMI, body mass index; NLR, neutrophil-to-lymphocyte ratio; TC, total cholesterol; TG, triglyceride; HDL-C, high-density lipoprotein cholesterol; LDL-C, low-density lipoprotein cholesterol; HbA1c, glycosylated hemoglobin. # Chi-square test; * Student *t* test; ** Mann–Whitney U test.

Table 5 shows subgroup comparison of clinical features and nutritional biomarkers between sleep disturbances and normal sleep groups in PD. Compared with normal sleep group, the sleep disturbances group demonstrated older age, longer duration, higher H-Y stage, UPDRSIII score, lower MNA score, PDSS score, BMI, hemoglobin, total protein, albumin and prealbumin (all *p* < 0.05). PDSS data in PD patients with different H-Y stages was demonstrated in Table 3.

**Table 5:** Subgroup comparison of clinical features and nutritional biomarkers between sleep disturbances and normal sleep groups in PD.

Characteristic	Sleep disturbances group (*n* = 88)	Normal sleep group (*n* = 80)	*P*
Male (*n*)	50	43	0.690#
Age (year)	72.09 ± 7.69	67.20 ± 8.48	<0.001*
Duration (year)	3 (1, 4.75)	2 (1, 3.0)	0.009**
H-Y stage	3 (2, 3)	2 (2, 2.5)	<0.001**
UPDRSIII	32.31 ± 16.06	22.14 ± 10.34	<0.001*
LED (mg)	340.55 ± 217.47	299.22 ± 176.13	0.207*
MNA	22.63 ± 4.45	25.84 ± 2.46	<0.001*
PDSS	68.10 ± 9.70	110.94 ± 13.39	<0.001*
BMI	22.89 ± 2.90	24.28 ± 2.53	0.001*
Hemoglobin (g/L)	128.19 ± 13.08	135.09 ± 13.09	0.001*
NLR	2.52 ± 1.36	2.20 ± 0.82	0.060*
Total protein (g/L)	63.01 ± 5.21	66.21 ± 4.81	<0.001*
Albumin (g/L)	37.74 ± 3.30	66.21 ± 4.81	<0.001*
Prealbumin (mg/L)	222.13 ± 55.67	248.39 ± 44.11	0.001*
Uric acid (umol/L)	301.32 ± 73.33	315.59 ± 84.89	0.244*
TC (mmol/L)	3.94 ± 0.84	4.18 ± 1.11	0.118*
TG (mmol/L)	1.21 ± 0.75	1.39 ± 1.01	0.187*
HDL-C (mmol/L)	1.15 ± 0.29	1.16 ± 0.28	0.868*
LDL-C (mmol/L)	2.26 ± 0.70	2.41 ± 0.85	0.211*
homocysteine (umol/L)	15.14 ± 5.29	14.99 ± 5.79	0.852*
Vitamin B12 (pg/ml)	505.89 ± 310.18	474.36 ± 332.16	0.526*
Ferritin (ng/ml)	171.56 ± 122.06	140.19 ± 84.35	0.053*
Folate (ng/ml)	7.06 ± 3.93	6.61 ± 3.75	0.445*
HbA1c (%)	6.20 ± 0.89	6.28 ± 0.99	0.565*

H-Y stage, Hoehn-Yahr stage; UPDRSIII, Unified Parkinson’s Disease Rating Scale Part III; LED, levodopa equivalent dose; MNA, Mini Nutritional Assessment; PDSS, Parkinson’s Disease Sleep Scale; BMI, body mass index; NLR, neutrophil-to-lymphocyte ratio; TC, total cholesterol; TG, triglyceride; HDL-C, high-density lipoprotein cholesterol; LDL-C, low-density lipoprotein cholesterol; HbA1c, glycosylated hemoglobin. # Chi-square test; * Student *t* test; ** Mann–Whitney U test.

Table 6 shows logistic regression analysis of sleep disturbances risk in PD patients. The dependent variable was the occurrence of sleep disturbances in PD patients. The independent variables were identified as age, duration, H-Y stage, UPDRSIII score, MNA score, BMI, hemoglobin, total protein, albumin and prealbumin. Multivariate logistic regression analysis identified both H-Y stage and total protein as significant risk factors for sleep disturbances in PD patients.

**Table 6:** Logistic regression analysis of sleep disturbances risk in PD patients.

Variable	OR	95% CI	*P*
Age (year)	1.037	0.980–1.098	0.202
Duration (year)	1.091	0.928–1.284	0.291
H-Y stage	4.257	1.322–13.710	0.015
UPDRSIII	0.986	0.929–1.046	0.632
MNA	0.871	0.721–1.052	0.152
BMI	0.913	0.777–1.072	0.264
Hemoglobin (g/L)	0.969	0.936–1.004	0.082
Total protein (g/L)	0.903	0.817–0.997	0.043
Albumin (g/L)	1.049	0.838–1.312	0.679
Prealbumin (mg/L)	1.000	0.991–1.010	0.918

H-Y stage, Hoehn-Yahr stage; UPDRSIII, Unified Parkinson’s Disease Rating Scale Part III; MNA, Mini Nutritional Assessment; BMI, body mass index.

## 4 Discussion

In this study, we found that approximately 28.0% of PD patients exhibited unfavorable nutritional status, including 10.1% with malnutrition and 17.9% at risk of malnutrition, findings consistent with those reported by Kacprzyk et al., in which the MNA identified 11.1% of PD patients as malnourished and 23.9% as being at risk of malnutrition (6). Racial factor and economic level contributed to the difference in nutritional status in PD. Fu et al. (5) demonstrated the prevalence of malnutrition of PD differed in 13 countries/regions of Asia, Latin America, and Europe, and the prevalence of nutritional disorders was higher in developing regions than that in developed regions. Although several hypotheses have been proposed regarding the mechanisms underlying malnutrition and risk of malnutrition in PD (4–6), the exact pathophysiology remains unclear. Potential contributing factors include inadequate nutrient intake, impaired gastrointestinal absorption or disruption of the gut-brain axis, increased energy expenditure, the neurodegenerative process itself, and levodopa therapy (20). Motor symptoms such as tremor, rigidity, and bradykinesia can impair fine motor control required for self-feeding. Furthermore, movement disorders in PD are associated with elevated energy demands (4–6). Non-motor symptoms, including gustatory dysfunction, hyposmia, sialorrhea, constipation, delayed gastric emptying, depression, anxiety, and sleep disturbances may also contribute to reduced nutrient intake and increase the risk of malnutrition (21). In addition to clinical manifestations, dopamine replacement therapy may influence nutritional status. Common side effects of levodopa, such as anorexia, nausea, vomiting, and dyskinesia (which increases energy expenditure), can negatively affect dietary intake (4). Moreover, a low-protein diet is often recommended to enhance levodopa efficacy, but this may further compromise nutritional adequacy (22).

Malnutrition is associated with increased mortality, loss of independence, higher susceptibility to infections, prolonged hospitalization, and diminished quality of life (20). Despite its high prevalence and clinical significance, data on malnutrition specifically in PD populations remain limited. The MNA is a simple, non-invasive, and well-validated tool for assessing nutritional status in older adults (17). In 2008, Barichella et al. (23) first applied the MNA for malnutrition assessment in PD patients. Compared to other screening methods, the MNA has the advantage of identifying individuals at risk of malnutrition before significant changes in body weight, BMI, or serum albumin occur, thereby enabling timely interventions and potentially improving clinical outcomes (5). Yılmaz et al. (24) found the malnutrition rate detected by Geriatric Nutrition Risk Index (GNRI) was lower than MNA in PD. Additionally, the MNA can be used to monitor the effectiveness of nutritional interventions over time (5).

We observed that 88 (52.3%) PD patients experienced sleep disturbances, a prevalence slightly lower than previously reported values (8). Sleep disturbances not only impair nocturnal sleep quality but also affect daytime functioning and overall quality of life for both patients and caregivers. They may also exacerbate motor and cognitive deficits, highlighting the importance of accurate diagnosis and appropriate management of sleep-related issues in PD, and reinforcing the need to incorporate sleep assessments into routine clinical evaluations. The pathophysiology of sleep disturbances in PD is multifactorial, involving nighttime motor complications and adverse effects of pharmacological treatments (8–10). Disruption of dopaminergic pathways projecting to brain regions involved in sleep–wake regulation, such as the substantia nigra pars compacta, ventral tegmental area, thalamus, brainstem, and cortical areas, provides one explanation for the high prevalence of sleep disorders in PD (25). Dysfunction in other neurotransmitter systems, including cholinergic, noradrenergic, and serotonergic pathways, has also been implicated in sleep disturbances, particularly in relation to RBD (26, 27).

Few studies have investigated the relationship between nutritional status and sleep disturbances in PD. Dietary intake can significantly influence hormonal regulation, inflammatory status, and neurotransmitter levels, all of which may directly or indirectly contribute to sleep disorders (11). Conversely, sleep disturbances can lead to various daytime impairments, hormonal imbalances, and reduced appetite, ultimately resulting in poorer nutritional status (11). Ozturk et al. (28) suggested a potential association between MNA scores and the Pittsburgh sleep quality index (PSQI), possibly mediated through shared impacts on gustatory function in older adults. Wang et al. (21) reported that poor nutritional status was linked to higher PSQI scores in PD patients. In our study, 15 blood-based biomarkers related to nutritional status were evaluated. The inclusion of these biochemical markers enhances the accuracy of nutritional assessment, as they reflect recent dietary intake and metabolic status.

Regarding potential nutritional biomarkers, previous studies have identified older age, longer disease duration, advanced H-Y stage, higher UPDRSIII score, and lower BMI as probable predictors of malnutrition in PD (4–6, 29, 30). In our study, similar associations were observed in both the poor nutrition and sleep disturbances subgroups with respect to these clinical indicators. Furthermore, regression analyses preliminary demonstrated that H-Y stage was an independent risk factor for sleep disturbances in PD. Barichella et al. (23) also observed that nutritional status deteriorates progressively with the duration of PD. As the disease advances, heightened energy expenditure and progressive neurodegeneration may result in a negative energy balance. This imbalance stems from increased energy consumption, such as that caused by levodopa-induced dyskinesias, which is not adequately compensated by sufficient dietary intake, either quantitatively or qualitatively. Consequently, this contributes to malnutrition and impaired sleep quality (29, 30).

Albumin is one of the most extensively studied proteins for assessing nutritional status. A systematic review has supported serum albumin as a useful indicator of overall nutritional status in older adults in non-acute settings (31). Albumin, the most abundant plasma protein in the body, possesses antioxidant and anti-inflammatory properties that may confer neuroprotective effects (32). In this study, we found that PD patients, particularly those in the malnutrition and sleep disturbances subgroups, exhibited lower albumin levels. Similarly, serum prealbumin has also been widely used as a biochemical marker of malnutrition. Recently, Xie et al. (12) identified prealbumin as a significant risk factor for sleep disturbances in PD. Moreover, logistic regression analysis in our study preliminary identified total protein as a significant predictor of sleep disturbances in PD patients. Total protein, which includes both albumin and globulin, reflects both nutritional status and systemic inflammation. These findings underscore the importance of monitoring a combination of albumin, prealbumin, and total protein to comprehensively assess nutritional and sleep-related conditions and guide timely interventions.

Hemoglobin, TC, TG, HDL-C, and LDL-C have also been recognized as potential nutritional markers (31). In this study, PD patients in the poor nutrition subgroup showed significantly lower levels of hemoglobin and TG compared to those with normal nutritional status. Clinical evidence suggests that hypercholesterolemia may exert a protective effect against the development of PD (31). Substantial research indicates that inflammation may be an early event and play a critical role in PD pathogenesis (33). The NLR is commonly used as a marker of systemic inflammation. In our cohort, NLR was significantly lower in the poor nutrition subgroup than in the normal nutrition subgroup, suggesting a potential link between nutritional status and inflammatory response. Studies have shown that higher uric acid levels are associated with a reduced risk of developing PD and slower disease progression, likely due to its antioxidant and neuroprotective properties (34). Homocysteine, which is closely linked to deficiencies in vitamin B12 and folate, may exacerbate oxidative stress and contribute to PD progression (35). Lin et al. (36) reported that HbA1c was significantly correlated with total MNA scores in PD. Elevated HbA1c levels have been associated with faster motor decline and cognitive deterioration in PD in prior studies (37), implying that early nutritional interventions targeting glycemic control may help slow disease progression. However, no significant differences were observed between the poor and normal nutrition subgroups regarding uric acid, homocysteine, vitamin B12, ferritin, folate, or HbA1c, which may reflect limited statistical power or a restricted role of these biomarkers in this population. Further studies with larger sample sizes are needed to validate the utility of these blood markers as predictors of malnutrition risk.

This study has several limitations. First, the sample size was relatively small and recruited from a single center; therefore, multicenter studies with larger cohorts are warranted. Second, blood biomarkers were measured only at admission, and longitudinal monitoring of dynamic changes in these parameters could provide more comprehensive insights. Third, the MNA is primarily validated for older adults, yet a portion of our participants were younger than 65 years, potentially limiting its applicability. Fourth, sleep disturbances were assessed using the PDSS without confirmation by polysomnography in specific cases, which may affect diagnostic accuracy. Fifth, both nutritional status and sleep quality may be influenced by levodopa treatment, making it challenging to exclude pharmacological effects from the analysis. Lastly, nutritional status can be influenced by socioeconomic factors and regional dietary habits, highlighting the need for future large-scale studies to account for these variables.

In conclusion, malnutrition, risk of malnutrition, and sleep disturbances are prevalent among PD patients. Nutritional biomarkers such as total protein are significantly associated with sleep quality in PD. Monitoring these biomarkers may facilitate early detection of nutritional deficits, and timely, individualized dietary interventions may improve sleep outcomes and overall patient management. However, this study is inherently cross-sectional in nature; therefore, longitudinal research is recommended in future studies to more robustly establish associations.

## Data Availability

The original contributions presented in the study are included in the article/supplementary material, further inquiries can be directed to the corresponding author.
